# Teledermatology to Improve Access to and Quality of Skin Care in Eastern Indonesia

**DOI:** 10.4269/ajtmh.23-0218

**Published:** 2024-01-02

**Authors:** Fidelis J. Adella, Hapu Ammah, Gladys O. Siregar, Maria Harianja, Evivana S. Sundari, Rahmat Sagara, Nicolas Tarino, Raph L. Hamers, Claus Bøgh, Hardyanto Soebono, Marlous L. Grijsen

**Affiliations:** ^1^ Sumba Foundation, Sumba, Indonesia;; ^2^ Department of Dermatology and Venereology, Siloam Hospital, Kupang, Indonesia;; ^3^ Oxford University Clinical Research Unit Indonesia, Faculty of Medicine Universitas Indonesia, Jakarta, Indonesia;; ^4^ Centre for Tropical Medicine and Global Health, Nuffield Department of Medicine, University of Oxford, United Kingdom;; ^5^ Department of Dermatology and Venereology, Gadjah Mada University, Yogyakarta, Indonesia

## Abstract

Skin diseases are a major public health concern in Indonesia, although access to specialized care in remote areas is limited. We initiated a low-cost teledermatology service in Sumba, a remote island in eastern Indonesia. Eighteen healthcare workers (HCWs) at five primary healthcare centers received training to manage common skin diseases and submit clinical cases beyond their expertise to an online platform. Submitted cases were reviewed by at least one dermatologist. Diagnostic agreement between HCWs and dermatologists was calculated. The HCWs participated in a satisfaction survey 2 years after project initiation. Since October 2020, of 10,384 patients presenting with skin complaints in a 24-month period, 307 (3%) were submitted for a teledermatology consultation. The most frequent skin diseases were infections and infestations (*n* = 162, 52.8%) and eczematous (85, 27.7%) and inflammatory (17, 5.5%) conditions. Fifty-three patients (17.3%) were diagnosed with a neglected tropical skin disease, including leprosy and scabies. Dermatologist advice was provided within a median of 50 minutes (interquartile range, 18–255 minutes), with 91.9% of consultations occurring within 24 hours. The diagnostic agreement level between HCWs and dermatologists significantly improved over time, from 46.9% in the first 6-month period (κ = 0.45; 95% CI, 0.37–0.54) to 77.2% in the last 6-month period (κ = 0.76; 95% CI, 0.67–0.86; global *P* < 0.001). The HCWs reported that the teledermatology service was extremely/very useful in supporting daily practice (100%) and improved their knowledge of skin diseases tremendously/a lot (92%). Teledermatology can improve accessibility and quality of skin services in medically underserved areas, providing opportunities for scalability and knowledge transfer to frontline HCWs.

## INTRODUCTION

Skin diseases comprise one of the largest disease burdens worldwide, affecting around one third of the global population, causing significant discomfort, disfigurement and loss in quality of life.^1^ Despite this profound impact, skin diseases receive little attention, especially in remote areas in low- and middle-income countries (LMICs). In Indonesia, with a population of 275 million, 9.4% of the populace lives below or close to the national poverty line.^2^ Despite impressive economic growth over the past decades and the nation’s conspicuous goal to achieve universal health coverage, widespread poverty and inequality persist.^3^^–^^5^

Skin diseases are common in Indonesia and are mostly managed by frontline healthcare workers (HCWs) in community health centers.^6^ Many are, however, overburdened owing to high workload, competing priorities, and often lack specific training in diagnosing and managing common skin diseases.^7^ As in many LMICs, specialist medical doctors are rarely available in rural and remote communities.^8^^–^^10^ Whereas the capital city Jakarta has an average of 60.2 specialist doctors per 100,000 population, Nusa Tenggara Timur (NTT), one of the country’s least developed provinces in eastern Indonesia, has 6.9 specialists per 100,000 population.^11^

Teledermatology has been shown to be a powerful educational and clinical support tool in the diagnosis and management of skin diseases, particularly in LMICs.^12^^–^^14^ To help address the underrecognized burden of skin diseases in Sumba, a remote and economically underdeveloped island in NTT province, we initiated a low-cost, sustainable teledermatology service to improve access to dermatological care and develop a better understanding of the needs of the community. This report highlights our experiences and lessons learned in the first 2 years of this ongoing project.

## MATERIALS AND METHODS

### Design.

The teledermatology service, launched on October 8, 2020, is an ongoing collaboration between Universitas Gadjah Mada in Yogyakarta; the Sumba Foundation, a privately funded, nonprofit, nongovernment healthcare provider in Sumba; and the Oxford University Clinical Research Unit in Indonesia. The teledermatology services received permission from the district health offices of West and Southwest Sumba. The project was conducted according to the principles of the Declaration of Helsinki. The initiative was financially supported by DermLink.

### Setting.

**Figure 1.**
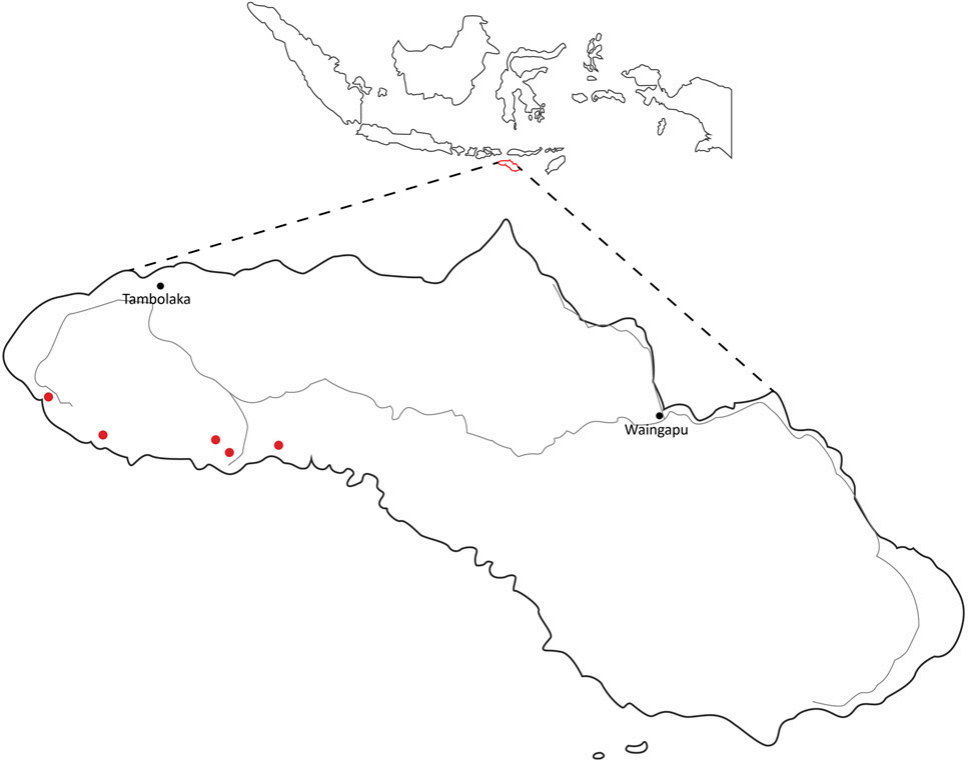
Map of Sumba Foundation clinics. The Sumba Foundation operates five primary healthcare clinics in West and Southwest Sumba. Circles from left to right: Waipakolo, Karang Indah, Lamboya, Hobawawi, and Hobajangi clinics.

Sumba, with a population of around 800,000, is plagued with high burdens of disease, and the availability, access to, and quality of health services are limited, with no dermatologist practicing on the island. In collaboration with the district health office, the Sumba Foundation has built and staffed five primary care clinics over the past decade (Figure 1). These clinics serve a catchment area of around 80,000 inhabitants and manage, free of charge, around 40,000 outpatient visits per year, of which ∼15% are skin related (Sumba Foundation annual report 2022, unpublished data).

### Teledermatology service.

Eighteen frontline HCWs employed by Sumba Foundation completed a 4-day clinical training program on the diagnosis and management of common and neglected skin diseases, delivered by three in-country dermatologists. The training included performing simple diagnostics such as a skin biopsy, skin scraping for scabies, potassium hydroxide for fungal skin infections, and slit-skin smears for leprosy. Subsequently, all frontline HCWs, including those who joined the Sumba Foundation in the following years, were invited to submit any clinical case that they considered beyond their confidence to a closed WhatsApp group that served as the tele-expertise platform. WhatsApp is a popular, free, end-to-end encrypted, mobile messenger application that is the predominant form of electronic communication in Indonesia.^15^ It is a simple and effective tool that is particularly useful in remote areas where internet access is often challenging. All Sumba Foundation staff own a smartphone that allows them to easily take pictures and immediately upload them through WhatsApp. Patients provided oral consent for taking photographic images and sharing them in the WhatsApp group. Submitters ensured that images, captions, and case descriptions did not contain any personal identifiers. Whenever possible, recognizable features (e.g., a tattoo) were masked using photo-editing software. Clinical diagnosis was based on the WHO *International Classification of Diseases* version 11 (ICD-11; https://icd.who.int/browse11/l-m/en). All submitted cases were reviewed by a team of (inter)national dermatologists with expertise in tropical infections, who provided diagnostic and therapeutic assistance with the shortest possible delay. The WhatsApp platform was also used for online training and sharing of information on specific topics.

### Data analysis and evaluation.

Each patient case submitted to the platform was counted as one consultation. The primary complaint was considered the primary diagnosis. The prevalent skin diseases were divided into nine categories based on the ICD-11. A data extraction form was designed in MS Excel. Data were extracted for all clinical cases and verified for completeness. Data were analyzed with R^®^ version 4.1.2 using descriptive statistics (R Foundation for Statistical Computing, Vienna, Austria). To measure inter-rater reliability, diagnostic agreement was calculated between HCWs (index) and dermatologists (reference standard) as proportions of agreement and Cohen’s kappa coefficient (κ). The κ is a metric used to assess agreement between two observers and can be interpreted as follows: no agreement (< 0.0) or slight (0.0–0.20), fair (0.21–0.40), moderate (0.41–0.60), good (0.61–0.80), very good (0.81 – < 1.0), or perfect (1.0) agreement. We also examined the level of diagnostic agreement 1) for the three largest disease categories (i.e., grouped as skin infections and infestations, eczematous conditions, and other conditions including papulosquamous and premalignant conditions, acneiform eruptions, toxic dermatitis, pigmented disorders and miscellaneous); and 2) changes over the successive 6-month time periods (χ^2^ test).

In September 2022, 2 years after inception of the project, we conducted an anonymous self-developed satisfaction survey among the HCWs who were part of the project. The online survey consisted of eight questions with a five-point Likert rating scale and was designed using REDCap software.

## RESULTS

### Staff and patient characteristics.

As of October 11, 2022, 24 months after the teledermatology services commenced, 307 clinical cases had been reviewed, an average of 12.8 patients per month. This comprised about 3% of the total of 10,384 patients who attended one of the Sumba Foundation health clinics for a skin complaint during the same time period. During this period, a total of 25 HCWs had access to the teledermatology platform: four were medical doctors (16.0%) and 21 were nurses (84.0%), with a median of 4.0 years (interquartile range [IQR], 2.0–6.0 years) of clinical experience. The median age of the HCWs was 28 (IQR, 28–32) years. Thirteen were female (52.0%). Currently, six of the 25 HCWs (24%) no longer work for the Sumba Foundation.

The median age of all patients reviewed was 19 (IQR, 3.0–44.5) years; range, 7 days to 89 years). Of all patients, 145 patients (47.2%) were below the age of 18 years, with 91 (29.6%) below the age of 5 years; 129 patients (42.0%) were female. Fourteen of 307 consultations (4.6%) were submitted via HCWs in government clinics outside the catchment area (Table 1). For each patient, two to four photographic images of sufficient quality were submitted to the platform.

**Table 1:** Sociodemographic characteristics of teledermatology consultations in Sumba, Indonesia

Characteristic	Consulted patients
	*N* = 307*
Female gender	129 (42.0)
Age (years), median (IQR)	19.0 (3.0–44.5)
Clinic location	
Hobawawi	132 (43.0)
Karang Indah	66 (21.5)
Lamboya	45 (14.7)
Waipakolo	44 (14.3)
Hobajangi	6 (2.0)
Other	14 (4.6)
Response time to consultation (minutes), median (IQR)	50 (18–255)
Response within ≤ 24 hours	282 (91.9)
Response within ≤ 48 hours	289 (94.1)

IQR = interquartile range.

* Data are listed as *n* (%) unless mentioned otherwise.

### Spectrum of skin conditions.

**Figure 2.**
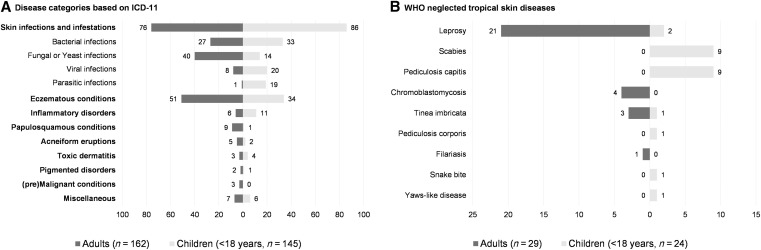
Distribution of skin diseases in Sumba, Indonesia. (**A** and **B**) Tornado plots showing the distribution of prevalent skin diseases (**A**) and neglected tropical skin diseases (**B**) among adults and children (< 18 years old) diagnosed through teledermatology in Sumba, Indonesia. In plot (**A**), the total number of patients with skin infections and infestations is shown, as well as the subdivision into bacterial, fungal, viral, and parasitic infections. ICD-11 = WHO International Classification of Diseases version 11.

Figure 2 provides an overview of the skin conditions diagnosed through teledermatology. A total of 83 different diagnoses were recorded (Supplemental Table 1). The largest group consisted of skin infections and infestations (162 consultations, 52.8%), comprising bacterial skin infections (60, 19.5%), fungal/yeast infections (54, 17.6%), viral infections (28, 9.1%), and parasitic infections (20, 6.5%). The second-largest group consisted of eczematous skin conditions (85, 27.7%), including atopic dermatitis (17, 5.5%), neurodermatitis (16, 5.2%), chronic eczema (15, 4.9%), acute eczema (12, 3.9%), hand eczema (5, 1.6%), seborrheic dermatitis (5, 1.6%), allergic (5, 1.6%) and irritant (3, 1.0%) contact dermatitis, and others (7, 2.3%). The third-largest group comprised inflammatory skin disorders (17, 5.5%), including persisting insect bites (6, 2.0%), urticaria (2, 0.7%), cutaneous lupus erythematosus (2, 0.7%), and others (7, 2.3%).

Most of the dermatoses identified were common skin diseases such as impetigo, tinea corporis, atopic dermatitis, neurodermatitis, and others. Less common diseases diagnosed included lichen nitidus, porokeratosis, metastasized acral lentiginous melanoma, ichthyosis, and a patient suspected of LEOPARD syndrome. Bacterial superinfections were seen in 35 of 273 patients (12.8%), excluding primary diagnosis with impetigo, ecthyma, abscess, vitiligo, lentigines, and hyperpigmentation. Fifty-three patients (17.3%) were affected by a neglected tropical skin disease (skin-NTD), including leprosy (23, 7.5%), scabies (9, 2.9%), pediculosis capitis (9, 2.9%), chromoblastomycosis, histopathology confirmed (4, 1.3%), tinea imbricata (4, 1.3%), pediculosis corporis (1, 0.3%), filariasis (1, 0.3%), and a snake bite (1, 0.3%). One child with skin ulcers was clinically suspected of having yaws (Supplemental Table 1).

### Teledermatology service.

The median time for a dermatologist to respond to a submitted case was 50 minutes (IQR, 18 minutes to 4 hours 15 minutes), ranging from less than 1 minute to a maximum of 9 days. The far majority of cases (282, 91.9%) were reviewed within 24 hours by at least one dermatologist, and 289 patients (94.1%) were evaluated within 48 hours, whereas for nine patients the response time was more than 4 days. The delays were mostly related to public holidays. In the majority of cases (*n* = 278, 90.6%), HCWs proposed a diagnosis and a treatment plan. Overall, the diagnostic agreement between the submitting HCW and the dermatologist was 56.7% (κ = 0.56; 95% CI, 0.50–0.61). Skin infections and infestations had the highest level of agreement between HCWs and dermatologists (67.9%; κ = 0.66; 95% CI, 0.59–0.73); the diagnostic agreement for parasitic infections was lower than that for bacterial, fungal/yeast, and viral infections (20.0% versus 75.0%, 81.5%, and 60.7%, respectively; no κ was available because of limited numbers). This was followed by eczematous conditions with 48.2% of agreement (κ = 0.44; 95% CI, 0.33–0.54). Over time, the level of diagnostic agreement increased significantly over successive 6-month time periods, from 46.9% in months 1–6 (κ = 0.45; 95% CI, 0.37–0.54; moderate) to 50.0% in months 7–12 (κ = 0.48; 95% CI, 0.33–0.63; moderate), 55.4% in months 13–18 (κ = 0.54; 95% CI, 0.41–0.67; moderate), and 77.2% in months 19–24 (κ = 0.76; 95% CI, 0.67–0.86; good) (Table 2; global *P* < 0.001). Of all consultations, only four (1.3%) required a hospital referral, and for 44 patients (14.3%) a follow-up consultation was reported.

**Table 2:** Diagnostic agreement between the submitting healthcare worker and the dermatologist*

Categories	Number of consultations	Full agreement (*n*, %)	No agreement (*n*, %)	Cohen’s κ coefficient (95% CI)	Level of diagnostic agreement
Overall					
All consultations	307	174 (56.7)	133 (43.3)	0.56 (0.50–0.61)	Moderate
Disease categories					
Skin infections and infestations	162	110 (67.9)	52 (32.1)	0.66 (0.59–0.73)	Good
Eczematous conditions	85	41 (48.2)	44 (51.8)	0.44 (0.33–0.54)	Moderate
Other conditions†	60	23 (38.3)	37 (61.7)	0.37 (0.25–0.49)	Fair
Time periods‡					
Months 1–6	130	61 (46.9)	69 (53.1)	0.45 (0.37–0.54)	Moderate
Months 7–12	42	21 (50.0)	21 (50.0)	0.48 (0.33–0.63)	Moderate
Months 13–18	56	31 (55.4)	25 (44.6)	0.54 (0.41–0.67)	Moderate
Months 19–24	79	61 (77.2)	18 (22.8)	0.76 (0.67–0.86)	Good

* Diagnostic agreement was calculated over all consultations, for three disease categories, and for each successive 6-month time period.

† Other conditions include papulosquamous and premalignant conditions, acneiform eruptions, toxic dermatitis, inflammatory and pigmented disorders, and miscellaneous.

‡ Global *P* value < 0.001, indicating a statistically significant increase in the level of diagnostic agreement over the 24-month period.

### Satisfaction survey among HCWs.

All 25 HCWs responded to the survey, including six who were no longer employed by the Sumba Foundation (Supplemental Table 2). The HCWs reported that the teledermatology service was extremely useful (22, 88.0%) or very useful (3, 12.0%) in supporting daily clinical practice. Almost half of the HCWs submitted a consultation at least once a month (48.0%), 10 sometimes (40.0%), 2 rarely (8.0%), and 1 (4.0%) never, as this HCW had recently joined the team. Most reported the teledermatology services improved their knowledge of skin diseases tremendously (13, 52.0%) or a lot (10, 40.0%). The dermatologists’ response time was considered adequate by 14 HCWs (56.0%), fast or very fast by 5 HCWs (20.0%), and slow by 6 HCWs (24.0%). The HCWs commented that they felt more competent diagnosing skin diseases and prescribing targeted therapy instead of prescribing multiple drugs to cover a wide variety of diagnoses and prescribing topical antibiotic or steroid cream instead of systemic drugs.

## DISCUSSION

Skin diseases were found to be a very common reason for attending outpatient services at the Sumba Foundation primary health clinics, with infectious conditions accounting for more than 50% of consulted visits. Three percent of all patients presenting with a skin complaint were submitted to the teledermatology platform. More than 90% of submitted cases received a dermatologist’s advice within 24 hours, and in one third of the submitted consultations this advice led to a change in diagnosis. During the course of the project, the level of diagnostic agreement between the HCWs and dermatologist significantly improved, increasing from 47% to 76% providing evidence that the teledermatology expert support and guidance consistently improved the diagnostic competence of the HCWs. The teledermatology service contributed to a decline in prescribing systemic antibiotic drugs, mitigating potential side effects and the risk of antimicrobial resistance. The HCWs improved dermatologic skills by providing better case descriptions, incorporating a (differential) diagnosis, and by mastering simple diagnostic techniques to corroborate their clinical judgment. Some HCWs also mentored their colleagues through the platform, commenting on the diagnosis and/or treatment plan, illustrating the potential for local capacity building and knowledge transfer. Overall, the HCWs were very satisfied with the teledermatology support, which boosted their knowledge and competence. Lastly, the teledermatology service also attracted referred patients from outside the catchment area, illustrating the wider need and impact of this health service.

The teledermatology service highlighted a significant burden of unrecognized, underreported skin diseases, warranting further public health action and research. We also found a significant number of patients with newly diagnosed and previously unrecognized skin-NTDs, such as leprosy, suggesting that this disease may be more prevalent in Sumba than previously thought. This important finding has triggered targeted active case finding efforts in the wider community to improve early diagnosis and treatment and prevent lifelong disabilities and community transmission.

The Government of Indonesia is making concerted efforts to provide universal health coverage through a national health insurance scheme (Jaminan Kesehatan Nasional).^4^ Nonetheless, challenges remain in delivering quality healthcare across the diverse and dispersed archipelago nation. Indeed, the impacts of skin diseases are often compounded by poverty, domestic crowding, poor hygiene practices, low level of education, malnutrition, climate (change), geographic isolation, and lack of access to healthcare services.^16^ By removing geographical and resource barriers, telemedicine provides an affordable and convenient tool to improve access to specialized care.^17^ Recent studies of teledermatology programs in French Guiana,^13^ Mali,^12^ Brazil,^18^ and Mongolia^14^ reported substantial reductions in unnecessary treatments, medical referrals, healthcare expenses, patients’ out-of-pocket costs, and travel distance.

We used WhatsApp, a widely used, free, instant messaging tool that is convenient and easily accessible in settings with limited internet connection.^19^^,^^20^ In previous reports, healthcare professionals acknowledged the importance of simple software that integrates well into a daily clinic with minimal efforts.^13^^,^^21^ WhatsApp is considered a safe platform, as no third party can intercept the information thanks to the default end-to-end encryption. However, legal, regulatory, and ethical concerns have been raised around potential data security issues in telemedicine.^19^^,^^20^ It is therefore fundamental that the smartphone be protected with a password and patient information be de-identified to ensure confidentiality. Additional security measures can be implemented in WhatsApp (i.e., two-step verification, switching off the automatic download function for images, and allowing messages to disappear to prevent storage on a user’s phone).^19^

The involvement of in-country dermatologists had several important benefits, such as their familiarity with endemic diseases, the local healthcare system and available resources, strengthening of local clinical capacities, absence of any language barriers, and being in the same time zone, which allowed for rapid responses. Although this teledermatology service is being maintained with relatively few resources, several challenges to its sustainability include the need for extended external funding to support training and diagnostic consumables, continuous investments in education given the high rate of staff turnover, and the long-term commitment of local dermatologists, warranting context-appropriate incentives.^21^ In addition, access to quality diagnostic pathology services is restricted in the area. To scale up the program, further investments are needed to build a robust, user-friendly, time-efficient, mobile platform suitable for information exchange with high-quality photos, follow-up, education, and research with the ability to leverage with other mobile technologies and artificial intelligence to aid in clinical decision support, already available in smartphone applications such as VisualDx and MyDermPath.^22^

In conclusion, we launched a low-cost, locally embedded teledermatology service that significantly improved the diagnostic competence of HCWs to manage skin diseases in a geographically isolated and medically underserved community in eastern Indonesia. The project demonstrated that, with limited resources, it is possible to empower frontline HCWs and improve access to specialized skin care. Local ownership, engaging in-country (tele)dermatologists, is key to successful capacity building and sustainability of the program.

## Supplemental files

10.4269/ajtmh.23-0218 Supplemental Materials
