# Supplementary material for: Teledermatology to Improve Access to and Quality of Skin Care in Eastern Indonesia
Source: Am J Trop Med Hyg. 2024 Jan 2;110(2):364–9. doi: 10.4269/ajtmh.23-0218 (PMC10859791; doi:10.4269/ajtmh.23-0218)
Supplement: Supplemental Materials [file tpmd230218.SD1.pdf]

Supplementary Material

Table S1. Spectrum of skin diseases reported through teledermatology in Sumba, Indonesia

	**Diagnosis**	**Number of patients (N, %)**
1	Impetigo	23 (7.5)
2	Tinea corporis	23 (7.5)
3	Multibacillary leprosy	22 (7.2)
4	Atopic dermatitis	17 (5.5)
5	Lichen simplex chronicus	16 (5.2)
6	Chronic eczema	15 (4.9)
7	Acute eczema	12 (3.9)
8	Herpes zoster	11 (3.6)
9	Pediculosis capitis	9 (2.9)
10	Scabies	9 (2.9)
11	Hand foot mouth disease	7 (2.3)
12	Hand eczema	6 (2.0)
13	Persistent insect bites	6 (2.0)
14	Pityriasis versicolor	6 (2.0)
15	Allergic contact dermatitis	5 (1.6)
16	Ecthyma	5 (1.6)
17	Seborrheic dermatitis	5 (1.6)
18	Tinea inguinalis	5 (1.6)
19	Candidiasis	4 (1.3)
20	Chromoblastomycosis	4 (1.3)
21	Dermatitis, non-specific	4 (1.3)
22	Folliculitis	4 (1.3)
23	Psoriasis	4 (1.3)
24	Psoriasis guttata	4 (1.3)
25	Tinea imbricata	4 (1.3)
26	Varicella	4 (1.3)
27	Diaper dermatitis	3 (1.0)
28	Irritant contact dermatitis	3 (1.0)
29	Paederus dermatitis	3 (1.0)
30	Pitted keratolysis	3 (1.0)
31	Cheilitis angularis	2 (0.7)
32	Chronic wound	2 (0.7)
33	Discoid lupus erythematosus	2 (0.7)

34	Folliculitis capitis	2 (0.7)
35	Pityriasis alba	2 (0.7)
36	Urticaria	2 (0.7)
37	Verruca vulgaris	2 (0.7)
38	Yeast infection	2 (0.7)
39	Abscess	1 (0.3)
40	Acneiform eruption induced by steroid abuse	1 (0.3)
41	Acral lentiginous melanoma	1 (0.3)
42	Actinic cheilitis	1 (0.3)
43	Balanitis	1 (0.3)
44	Bullous impetigo	1 (0.3)
45	Culicosis bullosa	1 (0.3)
46	Eczematous cheilitis	1 (0.3)
47	Filariasis	1 (0.3)
48	Herpes simplex	1 (0.3)
49	Hordeolum	1 (0.3)
50	Hyperpgimentation	1 (0.3)
51	Hypertrophic lichen planus	1 (0.3)
52	Ichtyosis	1 (0.3)
53	Intertrigo	1 (0.3)
54	Lentigines	1 (0.3)
55	LEOPARD syndrome (suspected)	1 (0.3)
56	Lichen nitidus	1 (0.3)
57	Marasmus	1 (0.3)
58	Miliaria	1 (0.3)
59	Miliaria pustulosa	1 (0.3)
60	Miliaria rubra	1 (0.3)
61	Mollusca contagiosum	1 (0.3)
62	Nummular eczema	1 (0.3)
63	Onychomycosis	1 (0.3)
64	Papular eczema	1 (0.3)
65	Paucibacillary leprosy	1 (0.3)
66	Pediculosis corporis	1 (0.3)
67	Peri-anal eczema	1 (0.3)
68	Pityriasis rosea	1 (0.3)
69	Porokeratosis	1 (0.3)
70	Prurigo	1 (0.3)
71	Prurigo nodularis	1 (0.3)
72	Pyoderma	1 (0.3)

73	Sebaceous hyperplasia	1 (0.3)
74	Snake bite	1 (0.3)
75	Tinea capitis	1 (0.3)
76	Tinea pedis	1 (0.3)
77	Toxic ertythema of the newborn	1 (0.3)
78	Verruca planae	1 (0.3)
79	Verruca plantaris	1 (0.3)
80	Vitiligo	1 (0.3)
81	Wound infection	1 (0.3)
82	Xerosis cutis	1 (0.3)
83	Yaws-like disease	1 (0.3)

Table S2. Satisfaction survey among 25 frontline healthcare workers participating in teledermatology services*

	1	2	3	4	5
1. Usefulness of teledermatology service in supporting daily practice	Not useful<br>-	A little bit useful<br>-	Moderately useful<br>-	Very useful<br>3 (12)	Extremely useful<br>22 (88)
2. Comfortable to submit a consultation in the WhatsApp group	Very uncomfortable<br>-	Uncomfortable<br>-	Neutral<br>2 (8)	Comfortable<br>12 (48)	Very comfortable<br>11 (44)
3. Frequency of submitting a consultation in the WhatsApp group	Never<br>1 (4)	Rarely (1x/year)<br>2 (8)	Sometimes (<1x/month)<br>10 (40)	Frequent (≥1x/month)<br>8 (32)	Very frequent (≥1x/week)<br>4 (16)
4. Improved my knowledge on skin diseases**	No improvement<br>-	A little bit<br>-	Moderately<br>1 (4)	A lot<br>10 (40)	Tremendously<br>13 (52)
5. Satisfied with the advice given by the dermatologists	Totally not satisfied<br>-	Not satisfied<br>-	Neutral<br>-	Satisfied<br>9 (36)	Very satisfied<br>16 (64)
6. Are recommendations adapted to local circumstances and resources?	Never<br>-	Seldom<br>-	Sometimes<br>6 (24)	Often<br>8 (32)	Always<br>11 (44)
7. Duration of response time by dermatologists	Too slow<br>-	Slow<br>6 (24)	Normal<br>14 (56)	Fast<br>4 (16)	Very fast<br>1 (4)
8. Recommend teledermatology for other clinics in remote settings	Very unlikely<br>-	Unlikely<br>-	Neutral<br>2 (8)	Likely<br>5 (20)	Very likely<br>18 (72)

The survey included eight questions using a 5-point rating scale. * Data are listed as n (%). ** 1 response was missing (participant commented (s)he had recently joined the group).
